# Genetic Variation and *De Novo* Mutations in the Parthenogenetic Caucasian Rock Lizard *Darevskia unisexualis*

**DOI:** 10.1371/journal.pone.0002730

**Published:** 2008-07-23

**Authors:** Tatiana N. Badaeva, Daria N. Malysheva, Vitaly I. Korchagin, Alexei P. Ryskov

**Affiliations:** Laboratory of Genome Organization, Institute of Gene Biology, Russian Academy of Sciences, Moscow, Russia; University of Uppsala, Sweden

## Abstract

Unisexual all-female lizards of the genus *Darevskia* that are well adapted to various habitats are known to reproduce normally by true parthenogenesis. Although they consist of unisexual lineages and lack effective genetic recombination, they are characterized by some level of genetic polymorphism. To reveal the mutational contribution to overall genetic variability, the most straightforward and conclusive way is the direct detection of mutation events in pedigree genotyping. Earlier we selected from genomic library of *D. unisexualis* two polymorphic microsatellite containg loci *Du281* and *Du215.* In this study, these two loci were analyzed to detect possible *de novo* mutations in 168 parthenogenetic offspring of 49 *D. unisexualis* mothers and in 147 offspring of 50 *D. armeniaca* mothers . No mutant alleles were detected in *D. armeniaca* offspring at both loci, and in *D. unisexualis* offspring at the *Du215* locus. There were a total of seven mutational events in the germ lines of four of the 49 *D. unisexualis* mothers at the Du281 locus, yielding the mutation rate of 0.1428 events per germ line tissue. Sequencing of the mutant alleles has shown that most mutations occur via deletion or insertion of single microsatellite repeat being identical in all offspring of the family. This indicates that such mutations emerge at the early stages of embryogenesis. In this study we characterized single highly unstable (GATA)_n_ containing locus in parthenogenetic lizard species *D. unisexualis*. Besides, we characterized various types of mutant alleles of this locus found in the *D. unisexualis* offspring of the first generation. Our data has shown that microsatellite mutations at highly unstable loci can make a significant contribution to population variability of parthenogenetic lizards.

## Introduction

Unisexuality in vertebrates has attracted wide attention since it was discovered. In squamate reptiles, unisexuality originates from interspecific hybridization between bisexual species and represents true parthenogenesis [1], [2]. They propagate via an aberrant gametogenetic mechanism that inhibits genetic recombination and causes clonal inheritance [1]. Hence the progeny consist of only genetically identical females with clonal inheritance in the next generation. Clonal reproduction and clonal diversity are the two features of unisexual vertebrates that make them attractive as model organisms in such areas as evolutionary ecology, genetics, cellular and molecular biology.

Parthenogenetic lizards of the genus *Darevskia* (formerly *Lacerta* [3]) were the first reptiles to be identified as unisexual [4]. Seven diploid all-female species are currently known, all from the Caucasus Mountains of Armenia [1], [5], [6]. Previous studies on these parthenogenetic species revealed some degree of allozyme variation [7]–[11] and low variability of mitochondrial DNA [12]. However multilocus DNA fingerprinting revealed very high levels of genetic variation in parthenogenetic populations of *Darevskia unisexualis*, *D. armeniaca*, *D. dahli* and *D. rostombecovi* [13]–[16]. The possible sources of such variation in parthenogenetic populations may be associated with multiple origins of clones from different pairs of founders, mutations, rare hybridization events, or some level of genetic recombination [8], [17]–[20]. However, the contribution of each of those events to the overall genetic variation remains unknown. The most straightforward and conclusive way to assess the mutational contribution to genetic variation is the direct detection of mutational events from pedigree genotyping [21]. Multilocus DNA fingerprinting with various microsatellite probes detected intrafamily variability of fingerprint patterns in *D. unisexualis* and *D. armeniaca* lizards [22], 23. These results imply that unstable loci may exist intheir genomes, but the real nature of such loci and supposed mutations remains obscure.

Recently Korchagin et al. (2007) cloned and sequenced a number of microsatellite loci of the parthenogenetic species *D. unisexualis*. Among several loci analyzed in detail only two, *Du281* (GenBank accession number AY 442143) and *Du215* (GenBank accession number AY 574928), which contain (GATA)_n_ repeats were polymorphic. However until now there was no information about genetical stability of those loci.

In the present work, *Du281* and *Du215* were tested in a pedigree based analysis to enable the detection of possible *de novo* mutations in parthenogenetic offspring of *D. unisexualis* and *D. armeniaca* lizards.

## Materials and Methods

Reproductively mature females of *D. unisexualis* and *D. armeniaca* were collected from natural habitats of western and central Armenia. The animals were maintained in separate enclosures in the laboratory until they began to produce eggs. The eggs were incubated under laboratory conditions. Genomic DNA was extracted from blood by standard phenol-chloroform extraction and resuspended in TE_buffer of pH 8.0. The loci *Du281* and *Du215* were amplified with the previously described primers (*Du281*: 5′TTGCTAATCTGAATAACTG3′, 5′TCCTGCTGAGAAAGACCA3′; *Du215*: 5′CAACTAGCAGTAGCTCTCCAGA3′, 5′CCAGACAGGCCCCAACTT3′) [24]. PCR reaction mix (20 µl) contained 20–40 ng genomic DNA, 1× PCR buffer (Dialat), 2 mM MgCl_2_, dNTP 0.25 mM each, and 0.625 units of *Taq*-polymerase (Dialat). Amplification conditions were: 94C for 3 min and then 40 cycles of 94C 1 min, 50C for 40 s, 72C for 40 s, followed by 72C for 5 min. The products, averaging about 200 bp in size, were separated by electrophoresis on a 8% native polyacrylamide gel (PAAG) and visualized on a ultraviolet light table following ethidium bromide staining. Amplified fragments were excised from the PAAG, purified and cloned into pMos blue vectors following standard procedures (pMos blueBlunt ended Cloning kit RPN 5110, Amersham Biosciences). The clones were amplified in MOSBlue competent cells grown at 37°C, and sequenced. The PCR products were cloned and sequenced using the chain termination reaction with ABI PRISM® BigDye™ Terminator v. 3.1 on an ABI PRISM 3100-Avant Genetic Analyzer.

The sequences of the allelic variants of the PCR products were compared using the MegAlign program (DNASTAR).

All animal procedures were carried out according to ethical principles and scientific standards of the ethical committee of Moscow State University.

## Results

**Figure 1:**
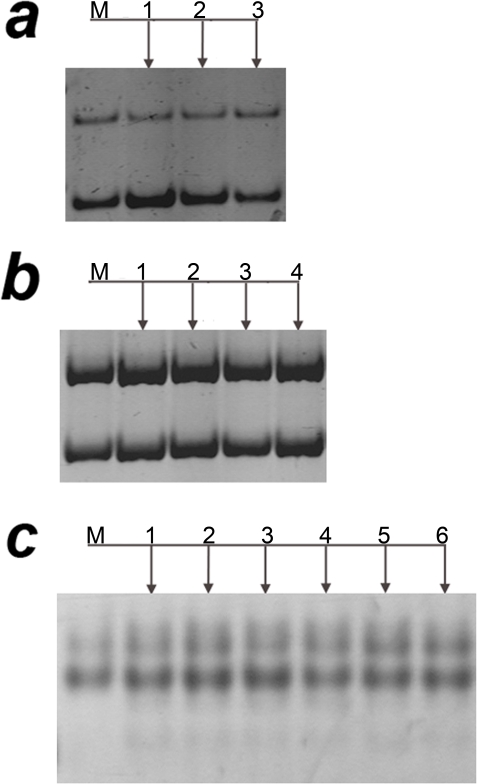
Examples of families where where no intrafamily variation of PCR products was revealed. a – Du281 locus, *D. armeniaca* family; b – 215 locus, *D. armeniaca* family; c – 215 locus, *D. unisexualis* family. Maternal DNAs are marked by M, offspring DNAs are shown by arrows and numbered in each family.

**Figure 2:**
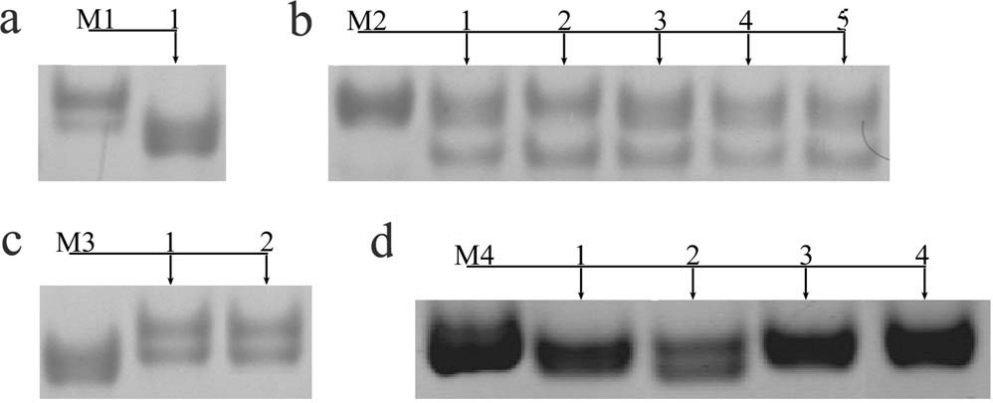
Intrafamily electrophoretic variability of PCR products amplified at *Du281* locus of parthenogenetic lizards *Darevskia unisexualis*. a – family 1, b – family 2, c – family 3, d – family 4. Maternal DNAs are marked by M1–M4 respectively; offspring DNAs are shown by arrows and numbered in each family.

In total DNA samples of 217 lizards (49 mothers and 168 offspring ) of *D. unisexualis* and 197 lizards (50 mothers and 147 offspring) of *D. armeniaca* were screened by locus-specific PCR. Mutant alleles were detected as changes in the electrophoretic mobility of PCR amplification products obtained from mother and their offspring. No mutant alleles were detected in *D. armeniaca* offspring at both *Du281* and *Du215* loci, and in *D. unisexualis* offspring at *Du215* locus. Figure 1 shows typical example of families where no intrafamily variation of PCR products was revealed. At the same time, 15 mutant alleles among offspring of four *D. unisexualis* mothers were found at the *Du281* locus (Fig. 2). These data show that the *Du281* locus of *D. unisexualis* is highly mutable, with an estimated mutation rate of 0.1428 events per germ line tissue.

To analyze the molecular structure of mutant alleles, we cloned and sequenced PCR products of the *Du 281* locus from maternal and mutant offspring of *D. unisexualis*. Comparison of nucleotide sequences of mothers and their offspring revealed mutations only in (GATA)_n_ microsatellite clusters, while no mutations were found in the flanking regions (Table 1). The haplotypes (T-A-T and C-G-C), formed by fixed point mutations in the flanking regions of microsatellite cluster, and specific for allelic variants of *Du281* [24] were used to mark maternal and corresponding offspring alleles. In family 1, consisting of the mother and one offspring, the deletion of one GATA monomer in microsatellite cluster was found in both offspring alleles. In family 2, consisting of the mother and five offspring, only one offspring allele marked by haplotype C-G-C was mutant with the deletion of GATATA in the microsatellite cluster of all offspring. In family 3, consisting of the mother and two offspring, an insertion of one GATA monomer was found in the microsatellite cluster of both offspring alleles. Family 4, consisting of the mother and four offspring, represent a more complicated case. While no mutations were observed among offspring alleles marked by haplotype T-A-T, different pattern of mutation was found among offspring alleles marked by haplotype C-G-C. In three offspring the mutatnt alleles revealed a deletion of one GATA monomer, but in another offspring a GATATA sequence was lost in the microsatellite cluster.

**Table 1:** Allelic variants of microsatellite clusters of *Du281* locus in parthenogenetic lizard families *D. unisexualis*.

Family 1.	
Maternal (M1) allele 1	•••T•••A•••(GATA)_9_ **(GATA)** GAT(GATA)TA(GATA)•••T•••
Offspring (1) allele 1	•••T•••A•••(GATA)_9_ - - - - GAT(GATA)TA(GATA)•••T•••
Maternal (M1) allele 2	•••C•••G•••(GATA)_9_ **(GATA)** (GATA)TA(GATA)•••G•••
Offspring (1) allele 2	•••C•••G•••(GATA)_9_ - - - - (GATA)TA(GATA)•••G•••
Family 2.	
Maternal (M2) allele 1	•••T•••A•••(GATA)_9_ GAT(GATA)TA(GATA)•••T•••
Offspring (1–5) allele 1	•••T•••A•••(GATA)_9_ GAT(GATA)TA(GATA)•••T•••
Maternal (M2) allele 2	•••C•••G•••(GATA)_9_ **(GATA)TA**(GATA)•••G•••
Offspring (1–5) allele 2	•••C•••G•••(GATA)_9_ **- - - - - -** (GATA)•••G•••
Family 3.	
Maternal (M3) allele 1	•••T•••A•••(GATA)_9_ - - - - GAT(GATA)TA(GATA)•••T•••
Offspring (1, 2) allele 1	•••T•••A•••(GATA)_9_ **(GATA)**GAT(GATA)TA(GATA)•••T•••
Maternal (M3) allele 2	•••C•••G•••(GATA)_9_ - - - - (GATA)TA(GATA)•••G•••
Offspring (1, 2) allele 2	•••C•••G•••(GATA)_9_ **(GATA)** (GATA)TA(GATA)•••G•••
Family 4.	
Maternal (M4) allele 1	•••T•••A•••(GATA)_9_ GAT(GATA)TA(GATA)•••T•••
Offspring (1, 3, 4) allele 1	•••T•••A•••(GATA)_9_ GAT(GATA)TA(GATA)•••T•••
Offspring (2) allele 1	•••T•••A•••(GATA)_9_ GAT(GATA)TA(GATA)•••T•••
Maternal (M4) allele 2	•••C•••G•••(GATA)_9_ **(GATA) (GATA)TA**(GATA)•••G•••
Offspring (1, 3, 4) allele 2	•••C•••G•••(GATA)_9_ **- - - -** (GATA)TA (GATA)•••G•••
Offspring (2) allele2	•••C•••G•••(GATA)_9_ (GATA) **- - - - - -** (GATA)•••G•••

Variations in microsatellite clusters are denoted by bold letters. T-A-T and C-G-C are haplotypes specific for allelic variants of *D. unisexualis* [24]. In Family 2 the observed changes were the same in all offspring (1–5). In family 3 the observed changes were the same in all offspring(1, 2). In Family 4 the observed changes are the same in three offspring (1, 3 and 4).

In summary, single repeat unit changes dominated (in microsatellite clusters of all offspring of the 1^st^ and 3^rd^ family and offspring 1, 2 and 4 of the 4^th^ family). In two families (all offspring of family 2 and offspring 2 of family 4) maternal and offspring microsatellite differed from each other by the deletion of GATATA imperfect monomer. Mutation may occur in both (1^st^ and 3^rd^ families) or only in one allele (2^nd^, 4^th^ and 5^th^ family). In three out of four families the patterns of mutations were similar in all offspring. The offspring of family 4 showed different pattern of mutations: in most individuals the mutant allele arose as a result of the loss of one microsatellite monomer, while in another it was the loss of imperfect monomer GATATA.

## Discussion

Unisexual vertebrates are useful model organisms for studying genome diversity because various mutational events can be easily detected in in pedigree genotyping. The genetic variation of the majority of such species is low in comparison with their sexual progenitors, and they face severe genetic and ecological restraints [2], [25]. Their genome and clonal diversity may arise as a result of mutations, multiple hybridization events, or some level of recombination occurring during continued clonal reproduction and the evolution of species [26]. In this study we characterized single highly unstable (GATA)_n_ containing locus in parthenogenetic lizard species *D. unisexualis*. Additionally, we characterized various types of mutant alleles of this locus found in the *D. unisexualis* offspring of the first generation. Comparison of maternal and offspring alleles of two polymorphic loci revealed *de novo* mutations only at the *Du281* locus in *D. unisexualis* offspring with the mutation rate of 0.1428 events per germ line tissue. This correlates with a higher level of population polymorphism in *Du281* in comparison with *Du215*. For instance, six and three allelic variants were detected among 65 *D. unisexualis* individuals for *Du281* and *Du215*, respectively [24]. According to Malysheva [22] only three allelic variants of the *Du215* locus were detected in *D. armeniaca* populations. The obtained mutation rate for *Du281* is comparable with the earlier results of DNA fingerprinting analysis. For instance, in *D. unisexualis* families the observed mutational rate was 0.9×10^−2^ per microsatellite band/per sibling when using a (GATA)_4_ hybridization probe [23]. These values are also within the range reported for individual microsatellite loci in bisexual species (from 10^−2^ to 10^−4^ per locus/per gamete) [21], [27]–[29]. For instance, pedigree analysis of mutations at human microsatellite loci gave estimates of mean mutational rates of 3×10^−3^–6×10^−4^ [21]. Genethon's extensive genotyping of >500 microsatellite loci in human population has suggested a lower mean genomic mutation rate, of 10^−4^ [30], [31]. A mutation rate of 5.7×10^−3^ was reported for (AAAG)_n_ tetranucleotide repeat locus in the barn swallow (*Hirunda rustica*) [27]. In the Australian lizard *Egernia stokesii* the mutational rate for (AAAG)_n_ locus was 4.2×10^−2^ [28]. A hypervariable microsatellite (TATC)_n_ with a mutation rate of 1.7×10^−2^ was found in the human X chromosome [32]. Some rodent-specific loci containing (GGCAGG)_n_ repeat motif showed germ-line mutation rates up to 8.8×10^−2^ per gamete [33].

Direct records of new length variants identified in comparisons between parents and offspring loci may be considered the most unambiguous way for analyzing mutational process in the germ line [27], [29]. Unisexual reptiles reproduce clonally and have a low level of recombinational events, thus all of the observed changes in microsatellite cluster are probably mutations. In three out of four *D. unisexualis* families the patterns of mutations were similar in all offspring, suggesting that mutation must be occurring in the mitotic creation of germ-line tissue, such that multiple oocytes would carry the same mutation.. The offspring of one family (offspring 3, family 4) showed different pattern of mutations: in most offsprings the mutant allele arose following the loss of a monomer, while in one offspring the imperfect monomer GATATA was lost. This offspring could mutate twice, first together with all other offspring and the second time at a later stage of differentiation, in turns, this mutation could have occurred once at the later stage of differentiation of germ line cells or even in a zygote. Studies of germ line microsatellite mutations, mainly in humans, found that mutations involving the gain or loss of a single repeat unit are much more frequent than multistep mutations [29]. In our findings we observed a mutation that occurred following the loss of an imperfect (GATATA) monomer, i.e. it involved more than one monomer. In other three families the changes of electrophoretic mobility were caused by the deletion/insertion of one GATA monomer, which fits with the stepwise mutation model [34].

Data from individual loci in several bisexual species [35] and pooled data on dinucleotide repeats in human genome [36], [37] show directionality in the mutation process, with an excess of insertions over deletions. On the contrary, our data showed a significant trend for mutation to lead to a decrease in allele size recorded in 11 out of 15 observed cases. This may be due to the peculiarities of structural organization of *Du281* locus, or to the specific features of the hybrid genome of *D. unisexualis*. Our data has shown that microsatellite mutations at highly unstable loci can make a significant contribution to population variability of parthenogenetic lizards.
